# Mild Endoplasmic Reticulum Stress Promotes Retinal Neovascularization via Induction of BiP/GRP78

**DOI:** 10.1371/journal.pone.0060517

**Published:** 2013-03-27

**Authors:** Shinsuke Nakamura, Haruka Takizawa, Masamitsu Shimazawa, Yuhei Hashimoto, Sou Sugitani, Kazuhiro Tsuruma, Hideaki Hara

**Affiliations:** Molecular Pharmacology, Department of Biofunctional Evaluation, Gifu Pharmaceutical University, Gifu, Japan; Children's Hospital Boston, United States of America

## Abstract

Endoplasmic reticulum (ER) stress occurs as a result of accumulation of unfolded or misfolded proteins in the ER and is involved in the mechanisms of various diseases, such as cancer and neurodegeneration. The goal of the present study was to clarify the relationship between ER stress and pathological neovascularization in the retina. Proliferation and migration of human retinal microvascular endothelial cells (HRMEC) were assessed in the presence of ER stress inducers, such as tunicamycin and thapsigargin. The expression of ER chaperone immunoglobulin heavy-chain binding protein (BiP), known as Grp78, was evaluated by real time RT-PCR, immunostaining, and Western blotting. Tunicamycin or thapsigargin was injected into the intravitreal body of oxygen-induced retinopathy (OIR) model mice at postnatal day 14 (P14) and retinal neovascularization was quantified at P17. The expression and localization of BiP in the retina was also evaluated in the OIR model. Exposure to tunicamycin and thapsigargin increased the proliferation and migration of HRMEC. Tunicamycin enhanced the expression of BiP in HRMEC at both the mRNA level and at the protein level on the cell surface, and increased the formation of a BiP/T-cadherin immunocomplex. In OIR model mice, retinal neovascularization was accelerated by treatments with ER stress inducers. BiP was particularly observed in the pathological vasculature and retinal microvascular endothelial cells, and the increase of BiP expression was correlated with retinal neovascularization. In conclusion, ER stress may contribute to the formation of abnormal vasculature in the retina via BiP complexation with T-cadherin, which then promotes endothelial cell proliferation and migration.

## Introduction

Angiogenesis is the formation of new blood vessels from a pre-existing vascular network, with continued expansion of a vascular tree [1], [2], [3]. In the retina, diseases that lead to this type of neovascularization are a major cause of blindness. Novel therapy for treatment of retinal neovascularization includes the use of anti-angiogenic substances such as anti-vascular endothelial growth factor (VEGF), which has shown great therapeutic efficacy [4]. However, the clinical needs are still unmet and many problems remain.

One difficulty in anti-angiogenic therapy is the unintended selective upregulation of other angiogenic factors that lead to resistance to anti-VEGF therapy [5], [6], [7], [8]. For example, intravitreal injection of bevacizumab cannot prevent rebleeding in eyes undergoing pars plana vitrectomy for treatment of diabetic vitreous hemorrhage. Another issue is the difficulty in providing continuous administration of anti-VEGF agents to the vitreous body, since the inhibition of retinal angiogenesis results in retinal ischemia and neovascularization reoccurs within a certain period of time [9]. Providing a basis for pathological vasculature-specific therapy requires exploration for the potential causative factor that gives rise to the development of pathological angiogenesis.

In this study, we focused on endoplasmic reticulum (ER) stress as a potent causative stress of angiogenesis. The ER is an essential cellular organelle where secretory and membrane proteins are synthesized and modified. It is also a major compartment for intracellular Ca^2+^ storage [10], [11]. ER stress arises as a consequence of accumulation of unfolded or misfolded proteins in the ER. This accumulation leads to the induction of immunoglobulin heavy-chain binding protein (BiP)/glucose regulated protein 78 (GRP78), followed by the activation of three ER-localized transmembrane proteins, including inositol-requiring enzyme 1α (IRE1α), PKR-like ER kinase (PERK), and activating transcription factor 6 (ATF6) [12], [13], [14], [15], [16]. These proteins are well known as sensors that interpret protein folding conditions in the ER and translate this information across the ER membrane to regulate downstream effectors; however, additional roles have now been reported.

Recent studies have revealed that conditional heterozygous knockout of *BiP* in the host endothelial cells results in severe reduction of tumor angiogenesis [17], and that IRE1α, PERK, and ATF6 mediate transcriptional regulation of *VEGF* under ER stress [18]. Excessive ER stress, which ultimately induces apoptosis, induces VEGF increases in both human retinal microvascular endothelial cells (HRMEC) [19] and murine retinas [20]. However, no definitive study has yet shown that ER stress actually induces retinal neovascularization in vasoproliferative retinopathies.

In the present study, we demonstrated, for the first time, that mild ER stress induced by low concentrations of tunicamycin (an antibiotic that blocks *N*-glycosylation of proteins) or thapsigargin (a specific inhibitor of intracellular sarcoplasmic reticulum Ca^2+^-ATPase -type Ca^2+^ pumps present in the sarcoplasmic/endoplasmic reticulum) promoted two critical angiogenic functions: endothelial cell proliferation and migration. We also showed that elevation of BiP expression on the cell surface and association of BiP with T-cadherin, a glycosylphosphatidylinositol (GPI)-anchored protein, were involved in proliferation and migration of HRMEC. *In vivo* administration of tunicamycin or thapsigargin also accelerated retinal neovascularization in a murine oxygen-induced retinopathy (OIR) model. Immunohistochemistry confirmed that the expression of BiP was involved in the formation of abnormal vasculature.

## Results

### ER Stress-induced Cell Proliferation in HRMEC

**Figure 1:**
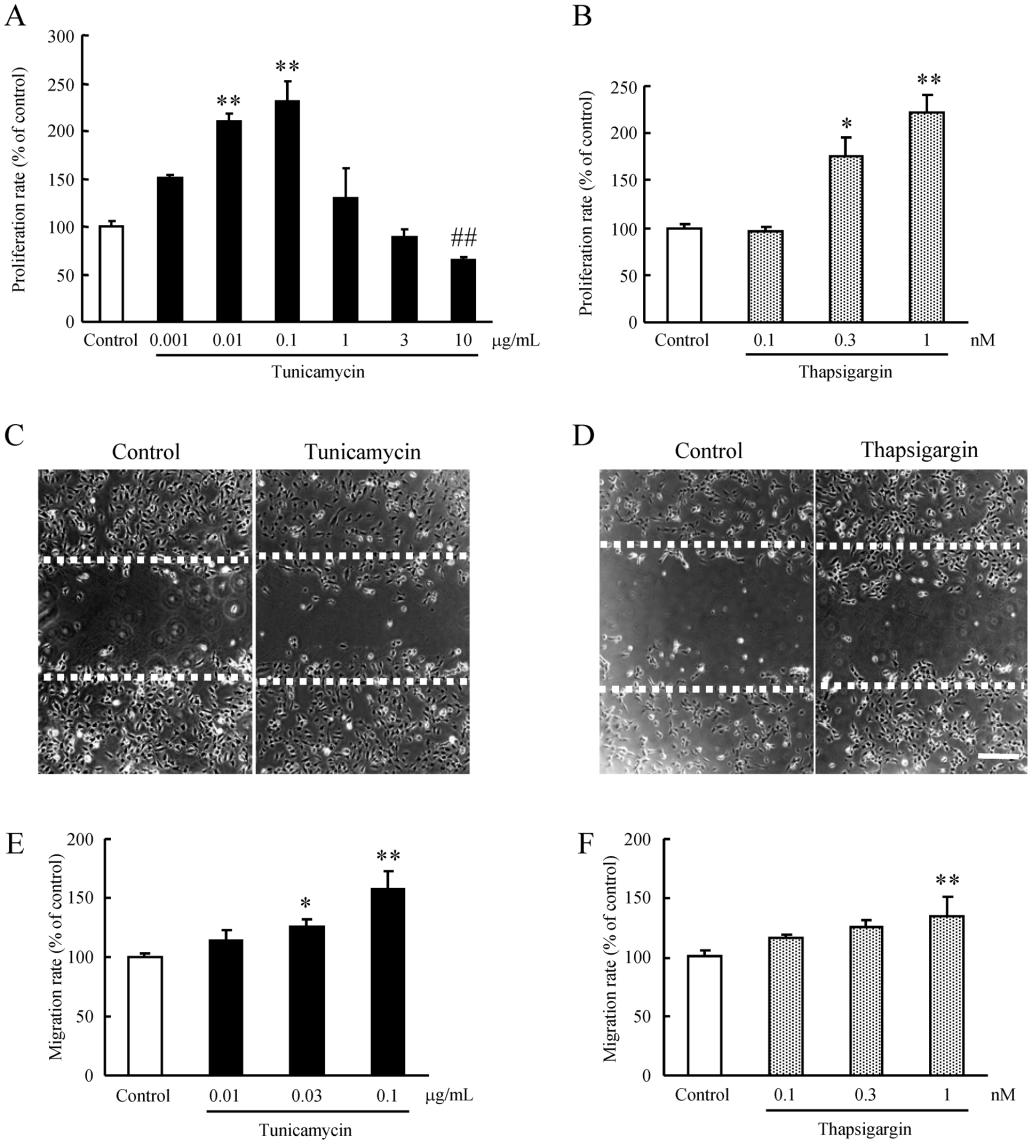
ER stress-induced proliferation and migration in HRMEC. HRMEC were cultured in a 96-well plate (at a density of 2×10^3^ cells/well), and were then supplemented with the indicated concentrations of (A) tunicamycin or (B) thapsigargin for 2 h, and measurements were made by WST-8 assay. Data are shown as mean ± S.E.M. (n = 6 or 12). *, p<0.05; **, p<0.01 vs. Control (Dunnett's multiple-comparison test). ##, p<0.01 vs. Control (Student's *t*-test). Migration of HRMEC was assessed using a wound-healing assay. Briefly, 90% confluent monolayers of HRMEC were scratch-wounded, and then incubated for 24 h. Images of the wounded monolayer of HRMEC were taken at 24 h after treatment for 2 h with (C) tunicamycin or (D) thapsigargin. Migration was estimated by measurement of cell numbers within the wounded region. The indicated concentrations of (E) tunicamycin and (F) thapsigargin increased migration compared to the control. Scale bars indicate 500 µm. Data are shown as mean ± S.E.M. (n = 3 to 7). *, p<0.05; **, p<0.01 vs. Control (Dunnett's multiple-comparison test).

We investigated the effects of ER stress in retinal microvascular endothelial cells by assessing the proliferation of HRMEC following treatment with several concentrations of the ER stress inducers tunicamycin and thapsigargin. Treatments with tunicamycin and thapsigargin at low concentrations (0.01, 0.1 µg/ml, and 0.3, 1 nM, respectively) resulted in a significant increase in proliferation of HRMEC (Fig. 1A and 1B). Cell proliferation by tunicamycin achieved a peak at 0.1 µg/ml (Fig. 1A).

### ER Stress-induced Cell Migration in HRMEC

We investigated whether ER stress induces migration of HRMEC by performing a wound-healing assay. The migration of HRMEC was significantly enhanced about 1.3- or 1.5- fold compared with the control in response to mild ER stress induced by low concentrations of tunicamycin (0.03 and 0.1 µg/ml) or thapsigargin (1 nM), respectively (Fig. 1C, 1D, 1E, and 1F).

### Severe ER Stress-induced Cell death in HRMEC

**Figure 2:**
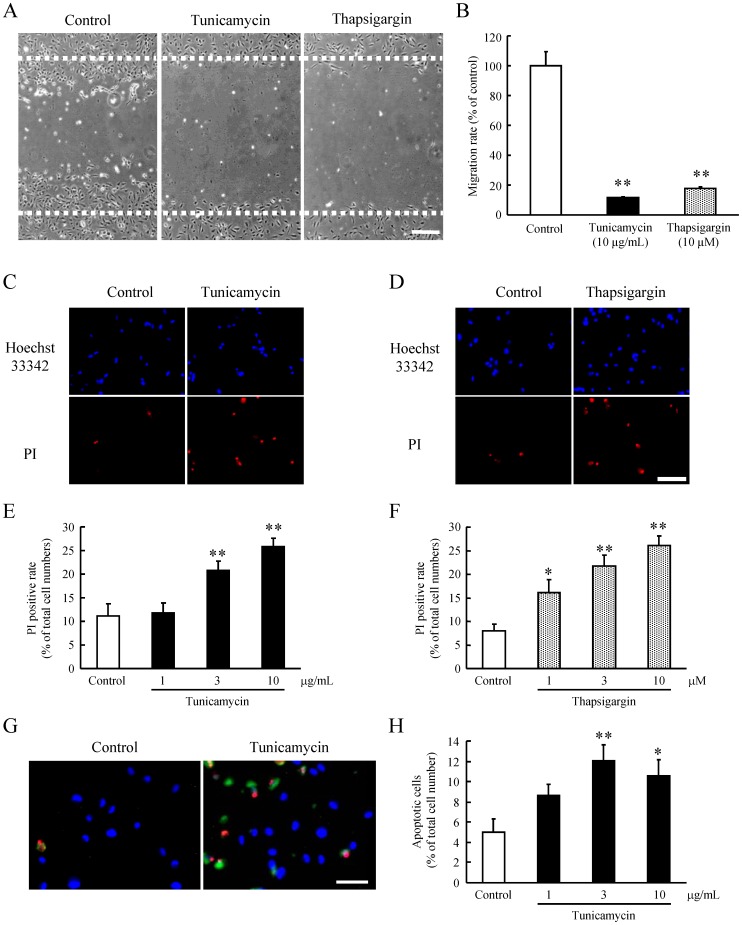
Severe ER stress-induced cell death of HRMEC *via* the apoptotis. (A) Representative images of migration test with tunicamycin at 10 µg/mL and thapsigargin at 10 µM are shown. (B) Both tunicamycin at 10 µg/mL and thapsigargin at 10 µM inhibited the cell migration in HRMEC. Fluorescence micrographs of Hoechst 33342 and PI staining are shown at 24 h after treatment for 2 h with (C) tunicamycin or (D) thapsigargin. Scale bar represents 100 µm. The dead cells were increased with (E) tunicamycin or (F) thapsigargin. Data are shown as mean ± S.E.M. (n = 6). *, p<0.05; **, p<0.01 vs. Control (Dunnett's multiple-comparison test). (G) Fluorescence photomicrographs of HRMEC triple-stained with propidium iodide (PI), annexin V-FITC, and Hoechst 33342, with tunicamycin. Red: Propidium Iodide, Green: Annexin V, blue: Hoechst33342. Scale bar represents 100 µm. (H) The apoptotic cells were significantly increased with tunicamycin at 3 and 10 µg/mL. Scale bar represents 100 µm. Data are shown as mean ± S.E.M. (n = 4). *, p<0.05; **, p<0.01 vs. Control (Dunnett's multiple-comparison test).

The migration of HRMEC was significantly reduced compared with the control in response to severe ER stress induced by a high concentration of tunicamycin (10 µg/ml) or thapsigargin (10 µM) (Fig. 2A and 2B). Severe ER stress by high concentrations of tunicamycin (3 and 10 µg/ml) and thapsigargin (1, 3 and 10 µM) induced cell death of HRMEC (Fig. 2C, 2D, 2E, and 2F). In the apoptotic cell death assay with annexin V-FITC staining, high concentrations of tunicamycin (3 and 10 µg/ml) induced the apoptosis in HRMEC (Fig. 2G and 2H).

### The morphological change of mild ER stress in HRMEC

**Figure 3:**
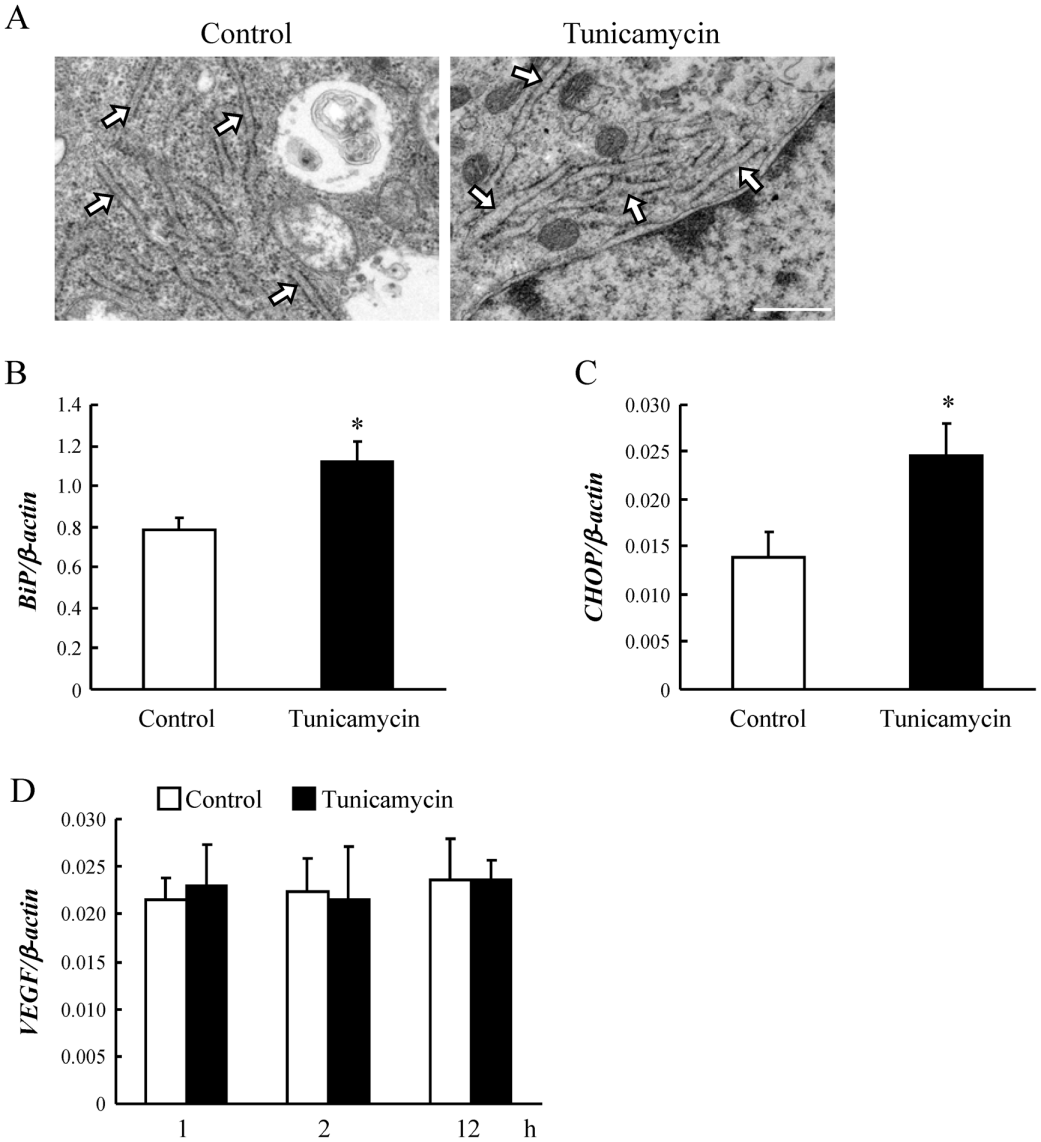
Tunicamycin at low concentration activated the transcription of *BiP* and *CHOP* mRNA with the mild dilation of ER in HRMEC. (A) Electron microscopic images are shown for 2 h with tunicamycin at 0.1 µg/mL. Tunicamycin induced the mild dilation of endoplasmic reticulum (ER). Scale bar represents 500 nm. Arrows indicate the ER in HRMEC. The mRNA levels of (B) *BiP* and (C) *CHOP* in HRMEC were determined by real-time RT-PCR and normalized against *β-actin*. Data are shown as mean ± S.E.M. (n = 4 to 6). *, p<0.05 vs. Control (Student's *t*-test). (D) *XBP-1* mRNA was not spliced by treatment with tunicamycin. HRMEC were harvested at 10 min, 30 min, 1 h, and 2 h after addition of tunicamycin, or at 10 and 22 h after incubation. Typical gel images of spliced or unspliced *XBP-1* bands at each sampling point were generated by MCE-202 MultiNA, a microchip based capillary electrophoresis system for DNA analysis (n = 3 or 4). (E) The mRNA level of *VEGF*, as a common angiogenic factor, was determined by real-time RT-PCR before and after the induction of *BiP* mRNA and *CHOP* mRNA, but no differences were apparent between the control and tunicamycin treated groups. Data are shown as mean ± S.E.M. (n = 4 or 5). For 1 h, p = 0.80; for 2 h, p = 0.91; for 12 h, p = 0.99 vs. Control (Student's *t*-test).

We investigated by observing the electron microscopy how the ER was changed in morphology by tunicamycin (0.1 µg/mL). Although the distinctive swelling of ER which causes cell death was not observed, tunicamycin at 0.1 µg/mL partially induced the morphological change of ER such as the swelling, compared to the control (Fig. 3A).

### ER Stress Promoted BiP and CHOP Gene Expression in HRMEC

We determined the nature of the factor involved in proliferation and migration induced by mild ER stress by exposing HRMEC to a low concentration of tunicamycin. The gene expression levels of representative unfolded protein response targets were then evaluated by real time RT-PCR. The mRNA expressions of *BiP* and *CHOP* were increased in HRMEC 2 h after treatment with tunicamycin (10 ng/ml) (Fig. 3B and 3C). On the other hand, no spliced form of *XBP-1* was observed after tunicamycin treatment (Fig. 3D). We also investigated the expression of *VEGF*, a major angiogenic factor, but no change was observed in the expression of *VEGF* mRNA in response to tunicamycin treatment (Fig. 3E).

### ER Stress Associated T-cadherin with BiP on the surface of HRMEC

**Figure 4:**
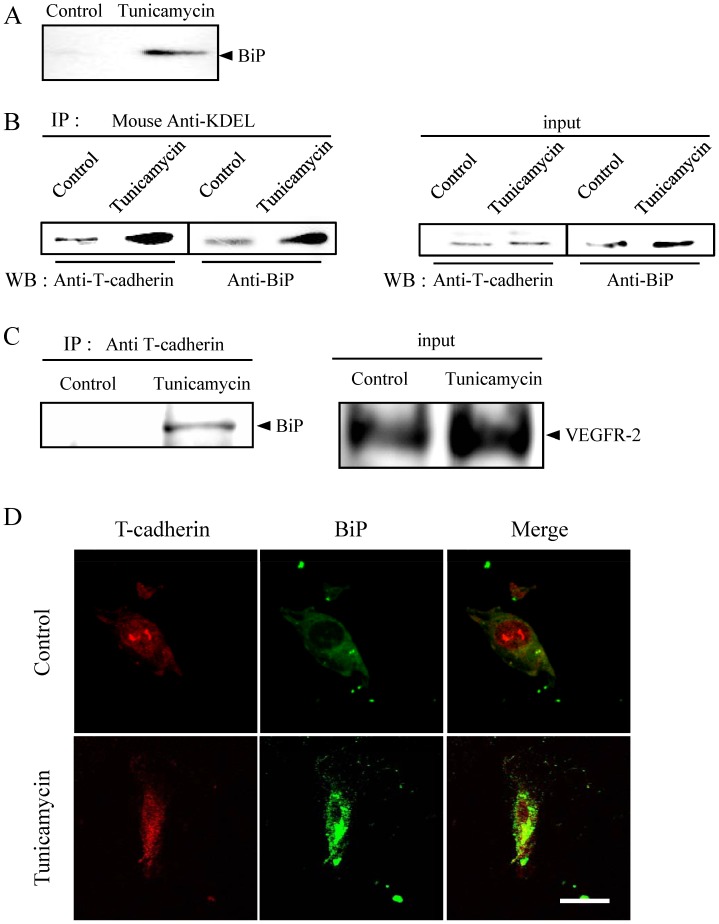
ER stress-induced formation of BiP/T-cadherin complexes on the surface of HRMEC. HRMEC were treated with 10 ng/ml of tunicamycin for 2 h. (A) BiP on the cell surface was precipitated after biotinylation to detect cell surface protein in untreated (control) or tunicamycin treated cells. (B) BiP and T-cadherin were immunoprecipitated with the anti-KDEL antibody to confirm the association of BiP with T-cadherin. Whole lysate without immunoprecipitation with the anti-BiP antibody was similarly evaluated. (C) After the membrane extraction, BiP and T-cadherin were immunoprecipitated with the anti-T-cadherin antibody and detected by using Western blotting with the anti-BiP antibody. Whole lysate without immunoprecipitation with the anti-VEGF receptor-2 (VEGFR-2) antibody was similarly evaluated to confirm the success of the membrane extraction. (D) Immunostaining showed the presence of BiP and T-cadherin on the surface of HRMEC. Scale bar indicates 30 µm.

Expression of BiP on the cell surface of proliferating endothelial cells has been reported [21], and cell surface BiP has been implicated in the induction of VEGF-independent angiogenesis [22]. On the other hand, glycosylphosphatidylinositol (GPI) -anchored T-cadherin is also reported to induce angiogenesis in a VEGF-independent manner [23], and this factor is also known to associate with BiP on the surface of vascular endothelial cells and influence endothelial cell survival [24]. We further examined the mechanism of VEGF-independent proliferation and migration by first measuring the expression of BiP on the cell surface using biotinylation of cell-surface protein followed by avidin agarose pull down and Western blotting. Western blots showed that cell surface BiP was upregulated by following treatment with 10 ng/ml tunicamycin for 2 h (Fig. 4A).

We then examined whether an interaction between BiP and T-cadherin was involved in proliferation and migration in HRMEC by performing co-precipitation and cell surface immunostaining of BiP and T-cadherin after a 2 h treatment with 10 ng/ml tunicamycin. Immunoprecipitation revealed an increased formation of a complex between BiP and T-cadherin in response to tunicamycin (Fig. 4B), although no similar increase was seen for T-cadherin before precipitation with anti-BiP antibody (Fig. 4B). Next, we performed the immunoprecipitation with anti-T-cadherin after extracting the plasma membrane to detect the interaction between BiP and T-cadherin in the cell surface. Mild ER stress induced by tunicamycin increased the formation of a complex between BiP and T-cadherin in the plasma membrane (Fig. 4C). We confirmed the extraction of plasma membrane by detecting the expression of VEGFR-2, serve as a marker for the cell surface with extract (Fig. 4C). Immunostaining of HRMEC with anti-BiP antibody and anti-T-cadherin antibody showed that BiP and T-cadherin were distributed in a diffuse manner on the cell surface and that they showed a tendency to further co-localize in the same area following tunicamycin treatment (Fig. 4D).

### ER Stress Accelerated Retinal Neovascularization in a Murine OIR Model

**Figure 5:**
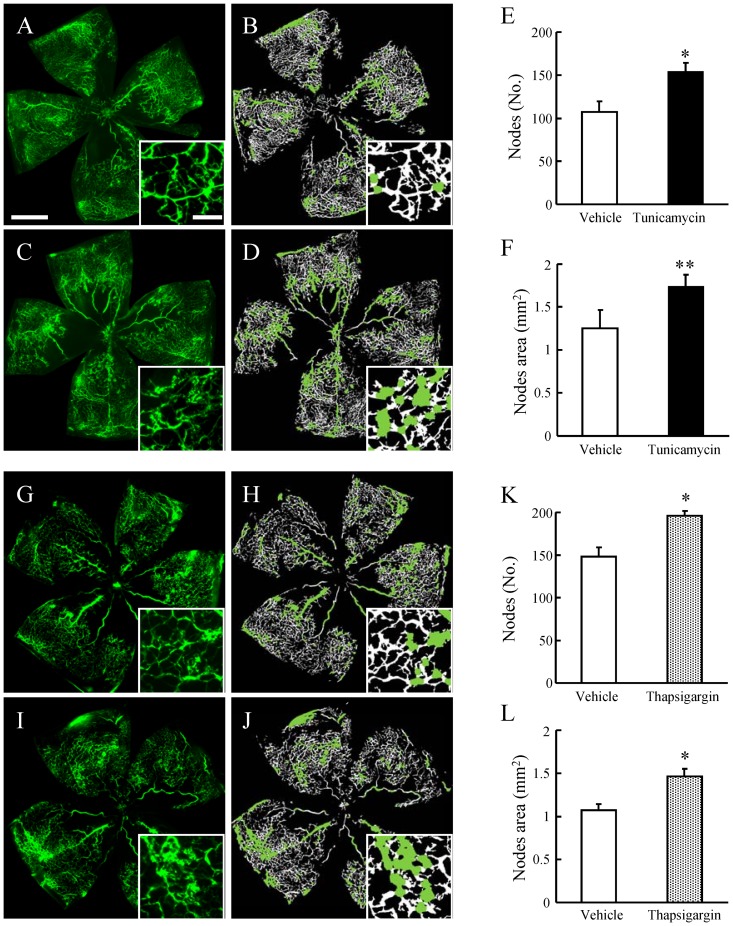
Tunicamycin and thapsigargin accelerated retinal neovascularization in a murine oxygen-induced retinopathy (OIR) model. The original images (A, C and G, I), together with the analyzed images (B, D and H, J) obtained using the Angiogenesis Tube Formation module in Metamorph, are shown. Scale bars indicate 500 µm and 100 µm (in box). Green labels in the analyzed images show the node regions. Quantitative analysis was performed on the entire retinal microvasculature in flat-mounted retinas obtained at P17. Tunicamycin at 3 µg/ml and thapsigargin at 1 µM increased (vs. vehicle) both the number of nodes (E, K) and the node areas (F, L), which are indexes of pathological neovascularization as calculated using the Angiogenesis Tube Formation module. Data are shown as mean ± S.E.M. (n = 5 to 9). *, p<0.05; **, p<0.01 vs.Vehicle (Student's *t*-test).

We evaluated the effect of ER stress on *in vivo* retinal neovascularization using the murine OIR model. Pathological vasculature (the node regions, represented by green labels in analyzed image) was increased by tunicamycin or thapsigargin treatment when compared with vehicle treatment (Fig. 5B, 5D, 5H, and 5J). The numbers and areas of nodes increased in both tunicamycin and thapsigargin treated groups (Fig. 5E, 5F, 5K, and 5L). The capillary free area, which was a result of hyperoxia treatment at P7–P12, was somewhat reduced in size in the tunicamycin-treated group (11.0±0.92%, mean ± S.E.M., n = 9, p = 0.24) when compared with the vehicle-treated group (13.6±2.01%, n = 7). The size of the capillary free area was significantly decreased in the thapsigargin-treated group (16.2±0.60%, n = 6, p = 0.002) compared with the vehicle-treated group (21.2±1.11%, n = 5).

The body weights of mice treated with tunicamycin or thapsigargin did not differ significantly from those of the vehicle-treated group. At P14, the body weights of vehicle-treated and tunicanycin-treated (3 µg/ml) mice were 4.24±0.28 (mean ± S.E.M., n = 7) and 4.05±0.30 (n = 9), respectively. At P17, these weights were 4.91±0.31 g and 4.66±0.27 g, respectively. The body weights at P14 of vehicle-treated and thapsigargin-treated (1 µM) mice were 3.98±0.25 (n = 5) and 4.18±0.15 (n = 6), respectively. At P17, these weights were 4.56±0.42 g and 4.77±0.24 g, respectively. Administration of tunicamycin or thapsigargin to the mice resulted in no statistically significant adverse effects on animal growth or development. The weight loss was not due to the influences of tunicamycin or thapsigargin because not only the drug-treated groups but also vehicle-treated group was low in the mice weight. Therefore, there are no problems to investigate the effects of tunicamycin or thapsigargin-treated groups compared to those of vehicle-treated group.

### ER Stress Increased BiP Expression in the Retinal Vasculature of a Murine OIR Model

**Figure 6:**
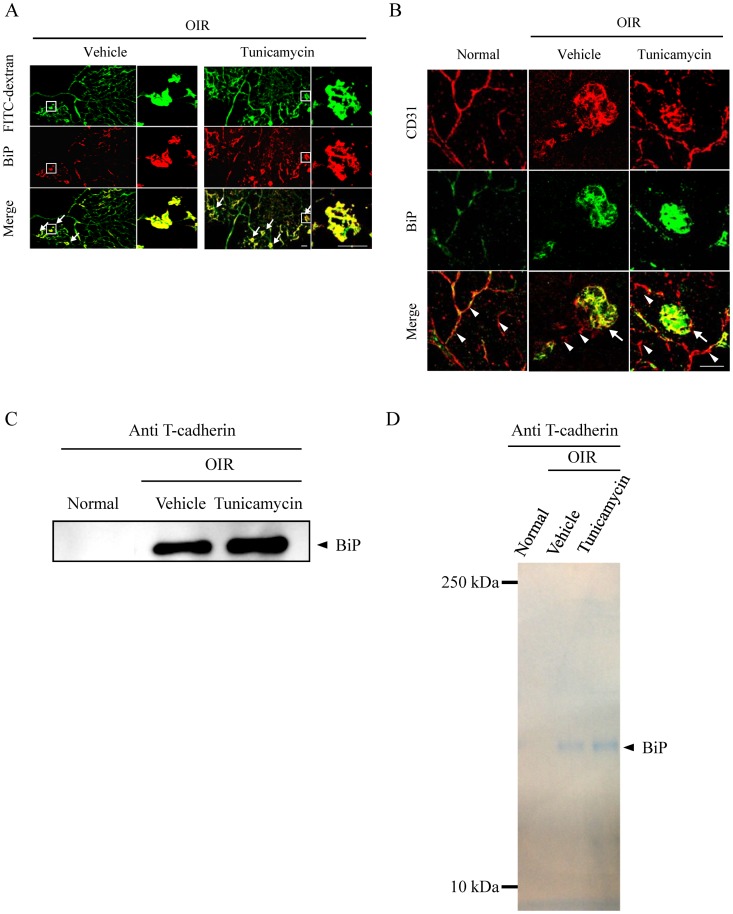
BiP was expressed in the retinal vascular endothelial cells of a murine oxygen-induced retinopathy (OIR) model. (A) OIR model mice at P17, treated with 3 µg/ml of tunicamycin at P14, were perfused with FITC-dextran and the retinas were stained with anti-BiP antibody. The original images (green channel) and BiP stained images (red channel) are shown, along with the analyzed images. (B) BiP (green channel) and CD31 (red channel) staining in flat-mounted retinas of P17 mice, under normoxia or subjected to the OIR protocol, following tunicamycin injection. Arrowheads and arrows indicate normal vessels and newly formed vessels anterior to internal limiting membrane, respectively. Scale bars indicate 50 µm. The complexes of BiP and T-cadherin in the retina were immunoprecipitated with the anti-T-cadherin antibody and detected by using Western blotting with the anti-BiP antibody (C), or by the Coomassie-staining (D).

We examined the induction of ER stress in the retina of OIR model mice and evaluated the effect of tunicamycin injection by first immunostaining the retinas of OIR mice with the antibody against BiP using OIR model mice following tunicamycin treatment. Immunostaining for BiP revealed that the expression of BiP following tunicamycin injection was localized in the retinal vasculature, and especially in the node regions (Fig. 6A). The localization of BiP in the retinal vasculature was further studied using double-immunofluorescence staining in normal and OIR model mice following tunicamycin injection. BiP was co-localized to microvascular endothelial cells (CD31) in the retina of both normal and OIR model mice (Fig. 6B). Furthermore, BiP was significantly expressed in the area of abnormal vasculature found in the retinas of OIR model mice, when compared with non-oxygen-treated controls. T-cadherin was linked with BiP in the retina of OIR model mice, and the connection protein level was increased by the administration of tunicamysin (Fig. 6C). An interaction between BiP and T-cadherin was specific to the retina in the OIR model by the coomassie-staining (Fig. 6D).

### The relationship between retinal neovascularization and BiP

**Figure 7:**
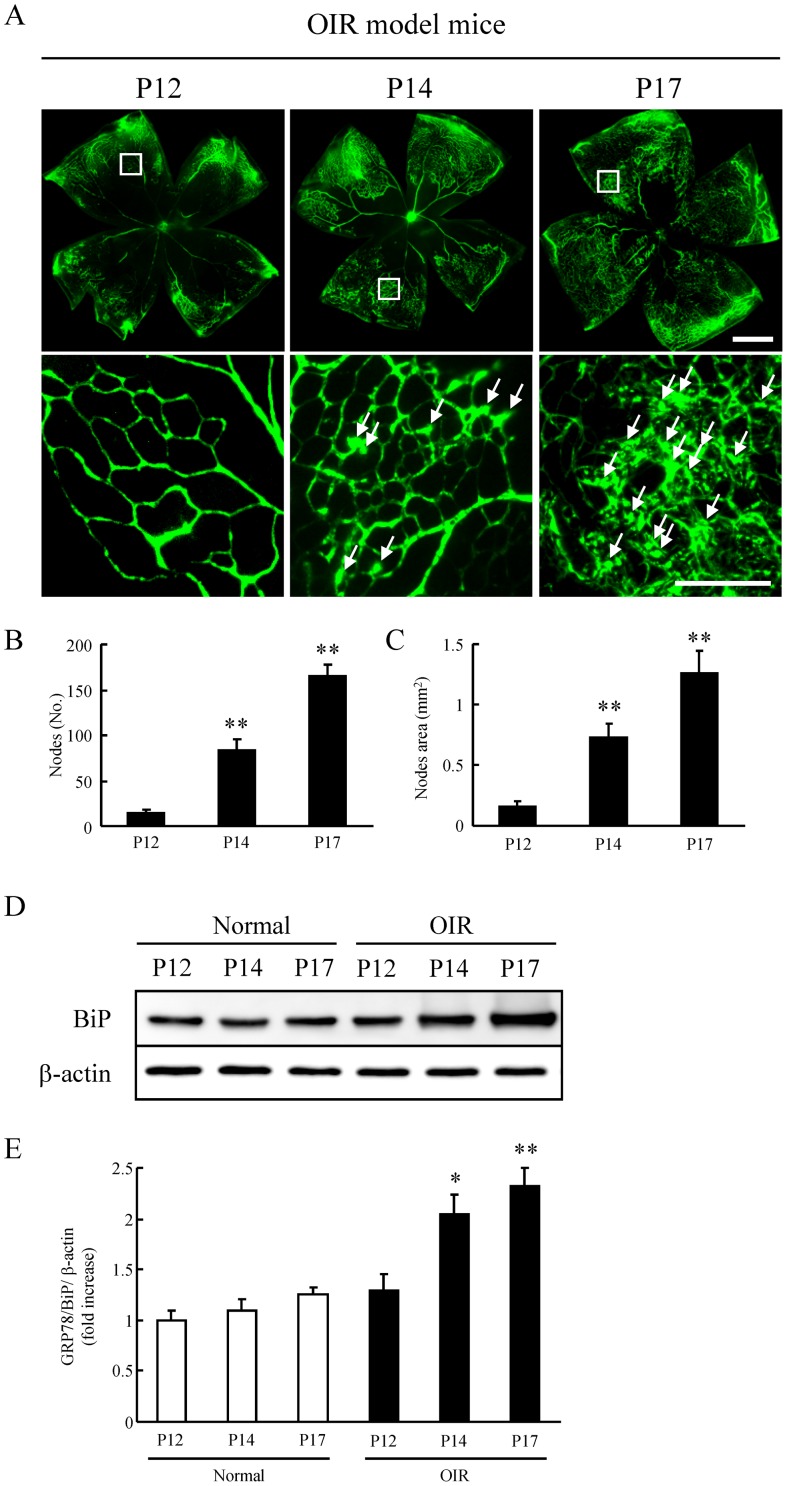
The expression of BiP was associated with retinal neovascularization of a murine oxygen-induced retinopathy (OIR) model. (A) The representative photographs are shown the retinal flat-mount (the upper images) and the higher magnification version of part of the corresponding upper images (the lower images). Arrows indicate newly formed vessels anterior to internal limiting membrane. Scale bars indicate 500 µm (Upper images) and 100 µm (Lower images). Quantitative analysis was performed on the entire retinal microvasculature time-dependently. In the OIR model, retinal neovascularization at P14 and P17 significantly increased both the number of nodes (B) and the nodes area (C), compared to that at P12. In the retina of the same individual, BiP induction was caused time-dependently (D). Relative intensity was described by the intensity of BiP divided by β-actin (E). Data are shown as mean ± S.E.M. (n = 6 to 9). *, p<0.05, **, p<0.01 vs. OIR mice at P12 (Dunnett's multiple-comparison test).

Retinal neovascularization was induced time-dependently in the OIR model. Both the nodes and nodes area in P14 and P17 OIR retinas showed marked increase versus control (Fig. 7A, 7B, and 7C). Furthermore, we investigated the interaction between the retinal neovascularization and the BiP expression by using the retinas of the same individuals. Although there is no change in the normal mice, BiP expression in the OIR model significantly increased at P14 and P17 (Fig. 7D and 7E).

**Figure 8:**
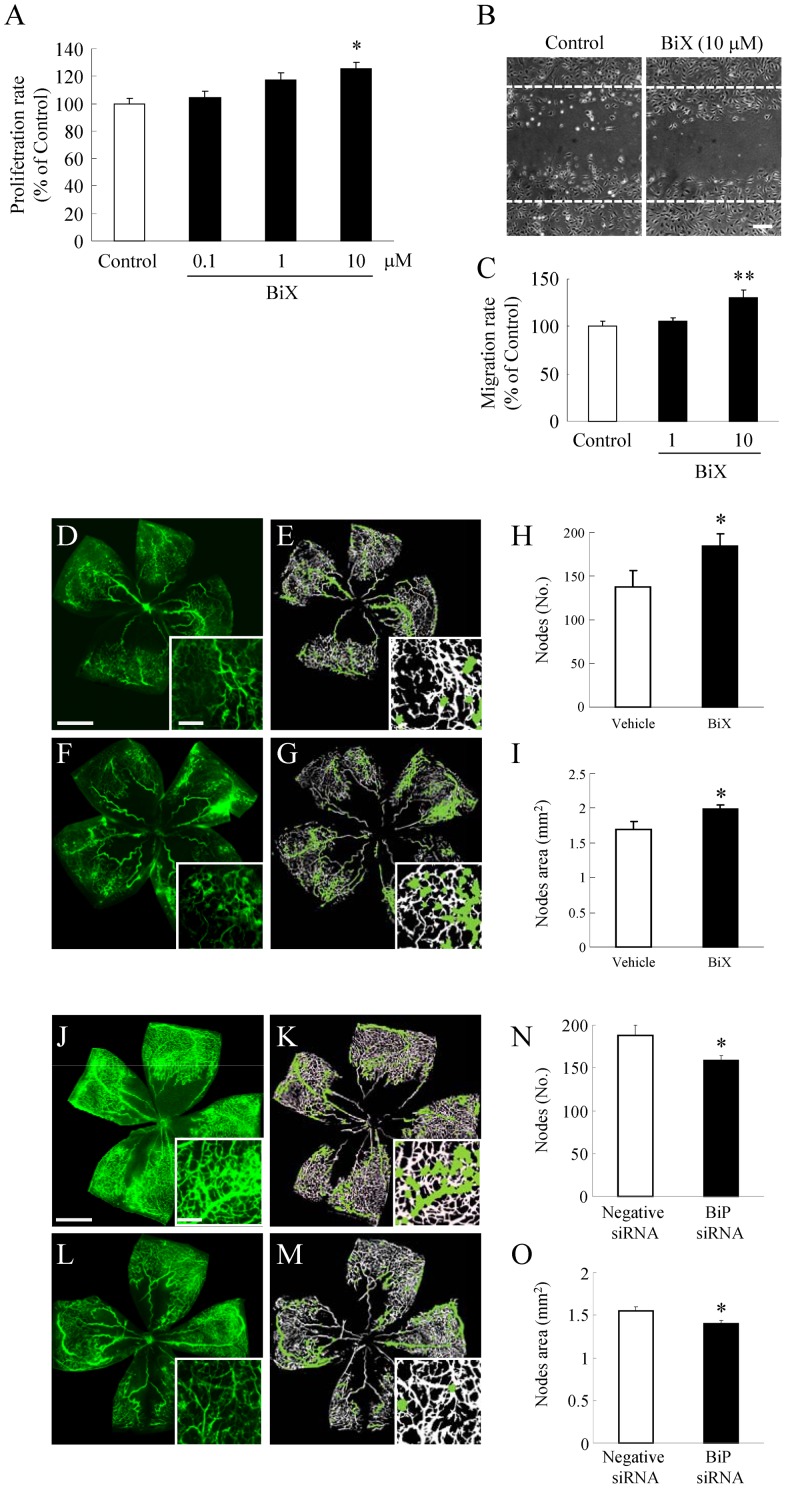
BiP protein levels regulates retinal neovascularization. (A) HRMEC were cultured in a 96-well plate (at a density of 2×10^3^ cells/well), and were then supplemented with the indicated concentrations of BiX for 1 h, and measurements were made by WST-8 assay. Data are shown as mean ± S.E.M. (n = 6). *, p<0.05 vs. Control (Dunnett's multiple-comparison test). Migration of HRMEC was assessed using a wound-healing assay. (B) Images of the wounded monolayer of HRMEC were taken at 24 h after treatment for 1 h with BiX. (C) The indicated concentrations of BiX increased migration compared to the control. Scale bars indicate 500 µm. Data are shown as mean ± S.E.M. (n = 4). **, p<0.01 vs. Control (Dunnett's multiple-comparison test). The original images (D, F and J, L), together with the analyzed images (E, G and K, M) obtained using the Angiogenesis Tube Formation module in Metamorph, are shown. Scale bars indicate 500 µm and 100 µm (in box). Quantitative analysis was performed on the entire retinal microvasculature in flat-mounted retinas obtained at P17. BiX at 1 µM and BiP small interfering (si) RNA at 1 µg/mL increased (vs. vehicle) both the number of nodes (E, K) and the node areas (F, L), which are indexes of pathological neovascularization as calculated using the Angiogenesis Tube Formation module. Data are shown as mean ± S.E.M. (n = 4 or 5). *, p<0.05 vs.Vehicle (Student's *t*-test).

Furthermore, we examined with BiP inducer X (BiX) or with BiP siRNA. Previously, we have reported that BiX preferentially induced BiP [25], [26]. BiX promoted the cell proliferation (Fig. 8A) and migration rates (Fig. 8B and 8C), and increased retinal pathologica neovascularization (Fig. 8D–I). Without tunicamycin or thapsigargin, we demonstrated that BiP could promote the retinlal neovascularization. Treatment of OIR mice with specific BiP small interfering (si) RNA significantly reduced neovascular outgrowth (Fig. 8J–O).

## Discussion

Ischemic retinal diseases, including diabetic retinopathy, retinopathy of prematurity, and age-related macular degeneration, are characterized by leaky vessels that grow in totally abnormal vasculature and damage the retina [27], [28]. The abnormal vessels cause retinal detachment and vitreoretinal hemorrhage, which lead to reduced visual acuity or visual field defects. The relationship between neovascularization and ER stress has recently been revealed; however, the actual role of ER stress in the formation of abnormal vasculature in the retina is unclear. In the present study, we verified that mild ER stress induced cell proliferation and migration, and accelerated retinal neovascularization. We also showed that BiP might be involved in the induction of this abnormal vasculature.

Proliferation and migration of vascular endothelial cells are a prerequisite for the angiogenic process [29]. In the present study, ER stress inducers triggered both proliferation and migration of HRMEC in a concentration-dependent manner. The concentrations of tunicamycin (0.01 and 0.1 µg/ml) and thapsigargin (0.3 and 1 nM) that indicates the acceleration of both proliferation and migration in the present study were from about one-fifty to one-thousandth of those (5 µg/ml and 1 µM, respectively) that have been reported to induce *VEGF* in mouse embryonic fibroblasts (MEFs) [18]. Therefore, the degree of ER stress induced by tunicamycin and thapsigargin in the present study was very mild compared with these earlier studies [18], [19].

A previous report has shown that tunicamycin at 1 µg/ml suppressed proliferation and migration in endothelial cells even in the presence of VEGF [30], indicating that mild ER stress may contribute to the pathology of vasoproliferative retinopathies. Rutkowski et al. demonstrated that survival of MEFs was favored at low concentrations of tunicamycin (25 ng/ml) or thapsigargin (2.5 nM) [31]. The low concentrations of tunicamycin and thapsigargin used in the present study also facilitated cell survival without induction of cell death, because we revealed that tunicamycin or thapsigargin induced the cell death through the apoptosis at more than 3 µg/mL and 1 µM, respectively. The mild ER stress response was accompanied by persistent BiP and transient CHOP protein expressions [31], which agreed with the results from real time RT-PCR.

On the other hand, previous reports have indicated that severe ER stress induced by high concentrations of tunicamycin (5 µg/ml) and thapsigargin (1 µM) resulted in endothelial apoptosis [32], [33], and promoted splicing of *XBP-1*. In this study, we indicated that tunicamycin at more than 3 µg/mL induced apoptosis in HRMEC. The spliced XBP-1 then bound to the *VEGF* promoter [18]. However, in the present study, we were unable to detect spliced *XBP-1* and *VEGF* mRNA levels were not increased by tunicamycin treatment. Additionally, the electron microscopic images are partially shown the ER dilation by the low concentrations of tunicamycin (0.1 µg/ml). Therefore, mild ER stress might promote retinal neovascularization by a different mechanism than that reported previously, where ER stress induced neovascularization *via* VEGF [18], [19], [20].

We focused on the VEGF independent-angiogenic pathway induced *via* BiP that was suggested by the results of real time RT-PCR. BiP is traditionally regarded as a major ER chaperone that facilitates protein folding; however, recent evidence indicates that BiP can also exist outside the ER. Cell-surface BiP has emerged as an important regulator of tumor cell signaling and viability as it forms complexes with a rapidly expanding repertoire of cell-surface protein partners, regulating proliferation, phosphoinositide 3-kinase (PI3K)/Akt signaling and cell viability [34], [35], [36]. In the present study, we showed that the expression of BiP on the cell surface of HRMEC and the formation of a BiP/T-cadherin immune complex were increased by treatment with tunicamycin.

T-cadherin (H-cadherin or cadherin-13) is an atypical member of the cadherin superfamily of adhesion molecules, and it is known to influence several parameters of angiogenesis including endothelial cell differentiation, migration, proliferation, and survival *via* activation of cyclin D_1_, Rho/Rac, and PI3K/Akt signaling pathways [37], [38], [39]. Since T-cadherin has been reported to promote angiogenesis in a VEGF-independent manner [23], and to associate with BiP in proliferating cells [24]. In fact, we suggested in the present study that T-cadherin bound to BiP specifically in the OIR model. The present results suggest that the immunocomplex formed between BiP and T-cadherin might be involved in the proliferation and migration in HRMEC and may operate *via* induction of BiP on the cell surface of HRMEC following mild ER stress.

We examined this potential involvement of BiP in retinal neovascularization by an *in vivo* study of the effect of ER stress on neovascularization. Tunicamycin and thapsigargin accelerated neovascularization in the retina of the OIR model mice. A previous report demonstrated upregulation of expression of BiP protein in the retina of OIR model mice at P14 [20]; however, the localization of BiP within the retina was unclear. The present study demonstrated that BiP was localized in retinal vascular endothelial cells and was highly expressed in node areas, which is an index of pathological neovascularization. On the other hand, no change in BiP localization was seen in the retinas of OIR mice, even following tunicamycin treatment. Both retinal neovascularization and BiP expression increased in the same individual. Taken together, these results suggest that the formation of abnormal vasculature might depend on BiP expression, which implicates BiP as a potential therapeutic target for vasoproliferative retinopathies.

We performed a series of *in vivo* studies using intravitreal injection of tunicamycin (3 ng/eye) or thapsigargin (0.65 ng/eye). The concentrations of tunicamycin and thapsigargin in the vitreous body after the intravitreal injections were estimated at approximately 600 ng/ml and 200 nM, respectively. The concentrations of tunicamycin and thapsigargin achieved within the retina in the present study will have been even lower than these. Based on our previous report, the estimated transition ratio of tunicamycin from the vitreous area to the retinal vasculature was considered to be about one-fiftieth [40], which suggests that the *in vivo* concentration of tunicamycin in the retina was very low and roughly similar to that employed in the present *in vitro* studies. Compared with the previous reports that used high concentrations of tunicamycin and induced VEGF in both HRMEC and murine retinas [19], [20], the present i*n vivo* study in a murine OIR model suggests that mild ER stress may contribute to retinal neovascularization *via* a VEGF-independent pathway. Our *in vitro* study results using HRMEC also support this possibility. In fact, we suggested that inducing BiP accelerated the retinal neovascularization and that the knockdown of BiP inhibited them. Without tunicamycin or thapsigargin, we demonstrated that BiP could promote the retinlal neovascularization.

In conclusion, the results presented here from both *in vitro* and *in vivo* studies reveal for the first time that BiP activation induced by mild ER stress might be involved in the formation of abnormal vascular networks. Mild ER stress may therefore serve to accelerate pathological angiogenesis in the retina.

## Materials and Methods

### Animals

The experimental designs and all procedures were approved by the Animal Experimental Committee of Gifu Pharmaceutical University (permission number; 2012-043). C57BL/6 mice (SLC, Shizuoka, Japan) were used. All mice were housed in a room with a 12-h light/dark cycle (light on at 8:00 a.m.) and had ad libitum access to food and water. All investigations were in accordance with the Association for Research in Vision and Ophthalmology statement for the Use of Animals in Ophthalmic and Vision Research, and the experiments were approved and monitored by the Institutional Animal Care and Use Committee of Gifu Pharmaceutical University.

### Cell Culture

Human retinal microvascular endothelial cells (HRMEC, DS Pharma Biomedical, Osaka, Japan) were cultured in a growth medium (CSC complete defined medium, DS Pharma Biomedical) at 37°C in a humidified atmosphere of 5% CO_2_ in air. The medium was supplemented with a defined cell boost. Subconfluent monolayers of HRMEC, from passages 3 to 8, were used in the experiments.

### Cell Proliferation

HRMEC were seeded into 96 well plate at a density 2×10^3^ cells/well at 37°C for 24 h in a humidified atmosphere of 5% CO_2_, and preincubated in CSC medium containing 10% fetal bovine serum (FBS) without cell boost at 37°C for 24 h. The HRMEC were supplemented with various concentrations of tunicamycin or thapsigargin for 2 h, and then washed with the medium was changed to the same (fresh) medium. Then, the HRMEC were incubated for a further 22 h. After incubation, the viable cell numbers were measured by means of a WST-8 assay. Briefly, 10 µl of CCK-8 (Dojindo, Kumamoto, Japan) was added to each well, incubated at 37°C for 3 h, and the absorbance measured at 492 nm.

### Wound-healing Assay

A wound-healing assay was performed to measure unidirectional migration. We modified part of the procedure described by our previous report [41]. HRMEC were seeded into a 12 well plate at a density 10×10^4^ cells/well at 37°C for 24 h in a humidified atmosphere of 5% CO_2_, and preincubated in CSC medium containing 1% FBS without cell boost at 37°C for 24 h. After 24 h incubation, the monolayers of HRMEC were scratched to a 1 mm depth in a straight line using a 10 to 200 µl micro-tip. After the HRMEC were reacted with tunicamycin or thapsigargin for 2 h, the medium was changed to the same (fresh) medium, and incubated for a further 22 h. Images of HRMEC in each well were taken at the time of the wounding and at 24-h intervals thereafter, using a phase-contrast microscope (Olympus, Tokyo, Japan). Migration was estimated by counting the cell numbers within the wounded region. Invading cells were counted by a single observer and taken as migrating cells. For each monolayer sample, four measurements were taken from four fields in each of independent wounds.

### Cell Death Assay

Under the same condition as a proliferation test, HRMEC were supplemented with tunicamycin for 2 h and incubated for a further 22 h. Cell mortality was measured using a single-cell digital imaging-based method employing fluorescent staining with fluorescent dyes (Hoechst 33342, Molecular Probes, Eugene, OR) and propidium iodide (PI; Molecular Probes). Images were collected using an Olympus IX70 inverted epifluorescence microscope (Olympus, Tokyo, Japan), as in a previous report [42].

Furthermore, we performed annexin V-FITC (Trevigen, Gaithersburg, MD) staining to detect apoptotic cell death by adding tunicamycin. Briefly, initially washed in PBS, were incubated with 1 µl of annexin-FITC and PI for 15 min at room temperature according to the manufacturer's instructions. Annexin-FITC/PI-stained samples were diluted in 400 µl of 1× annexin binding buffer. As previously described, the photographs were taken by using an Olympus IX70. Early apoptotic cells are defined as having annexin positive, PI negative staining. Late apoptotic and non-viable cells are both annexin and PI positive. The following equation was used to analyze apoptotic cells:

Apoptotic cells (%) = [annexin (+)+PI (−)+Hoechst (+)]/(total cells)×100

### Electron Microscopy

Under the same condition as proliferation test, HRMEC were supplemented with tunicamycin (0.1 µg/ml) for 2 h and incubated for a further 22 h. We retrieved HRMEC by using the cell scraper, and were pre-fixed wtih 2.5% glutaraldehyde for 2 h, followed by 1 h post-fixation with 1% osmium tetroxide (OsO_4_), both in 0.1 M sodium cacodyrate buffer (pH 7.4). They were dehydrated through a grated series of ethanol and were embedded in Epon 812. Ultra-thin sections were cut with Leica Ultracut-S (Leica Co. Ltd, Wetzlar, Germany) and directly mounted onto 400-mesh copper grids. They were stained on the droplet of 4% uranyl diacetate Gd (CH_3_COO)_2_) for 20 min at room temperature and rinsed thrice with several drops of distilled water.

Samples were subjected to observation under transmission electron microscope (JEM-1010, JEOL Ltd., Tokyo, Japan) at an acceleration voltage of 100 kV with an objective aperture of 20 µm. Photographs were taken with a 16-bit slow-scan CCD canera (1024×1024 pixels) at 5000× nominal magnification.

### Reverse Transcription (RT)-PCR

HRMEC were cultured at the same density of proliferation in a 12 well plate and incubated for 24 h at 37°C in 5% CO_2,_ and preincubated in CSC medium containing 10% FBS without cell boost at 37°C for 24 h. Then, HRMEC were supplemented with tunicamycin (0.1 µg/ml) for 2 h and incubated for a further 22 h. HRMEC were harvested at different time points after treatment with tunicamycin and then evaluated by MCE^®^-202 MultiNA (Microchip Electrophoresis System for DNA/RNA Analysis) or Thermal Cycler Dice Real Time System. After total RNA was extracted, real time RT-PCR was performed using Prime script RT reagent kit (Takara, Tokyo, Japan) and SYBR Premix EX Tq II (Takara). The mRNA levels of target genes were normalized against *β-actin*. Primers specific for *BiP* (forward, 5′-GCCTGTATTTCTAGACCTGCC-3′; reverse, 5′-TTCATCTTGCCAGCCAGTTG-3′), *CHOP* (forward, 5′-GACCTGCAAGAGGTCCTGTC-3′; reverse, 5′-TGTGACCTCTGCTGGTTCTG-3′), *VEGF* (forward, 5′-GACAAGAAAATCCCTGTGGGC -3′; reverse, 5′-AACGCGAGTCTGTGTTTTTGC-3′) and *β-acitin* (forward, 5′-CATCCGTAAAGACCTCTATGCCAAC-3′; reverse, 5′-ATGGAGCCACCGATCCACA-3′) were used in real time RT-PCR, and a specific primer for *XBP-1* (forward, 5′-GAAGCCAAGGGGAATGAAGTGAGG-3′; reverse, 5′-CATGGGGAGATGTTCTGGAGGGG-3′) was used in semi quantitative RT-PCR.

### Biotinylation of Surface Proteins

HRMEC in four 10 cm dishes at the same density of proliferation were preincubated in CSC medium containing 10% FBS without cell boost at 37°C for 24 h. After incubation, HRMEC were supplemented with tunicamycin (10 ng/ml) for 2 h and then washed twice with ice-cold phosphate buffered saline (PBS buffer). Selective labeling of cell-surface proteins was performed by incubation of HRMEC with 0.25 mg/ml impermeant EZ-Link^®^ Sulfo-NHS-SS-Biotin (Thermo Fisher Scientific, Waltham, MA, USA) for 30 min at 4°C. Then, the cells were scraped into solution, transfer the contents of four 10 cm dishes to a single 50 ml conical tube and centrifuge cells at 500× g for 3 min. Similarly treated cells without tunicamycin were prepared as a control. After discarded supernatant, cells were washed with tris-buffered saline (TBS buffer) solution and lysed with IP Lysis/Wash Buffer (Thermo Fisher Scientific) containing protein inhibitors. The lysate was divided by two and used for immunoprecipitation to purify surface proteins or to detect T-cadherin/BiP complexs.

### Immunoprecipitation

For the detection of cell surface expression of BiP, Neutravidin-agarose beads (Thermo Fisher Scientific) were added and mixed well with the lysate at 4°C for 1 h. The bounded proteins were released by incubating with SDS-PAGE sample buffer. For co-immunoprecipitation with BiP, the lysate was incubated with anti-KDEL antibody (Stressgen Biotechnologies, Victoria, BC, Canada) at 4°C for 24 h. After incubation, it was added to Protein A/G Plus Agarose (Thermo Fisher Scientific) in spin column and incubate further for 1 h. Immunocomplexes bound to Protein A/G Plus Agarose were pelleted and eluted. Immunoprecipitation products were analyzed by Western blotting using anti-BiP antibody (BD Biosciences, San Diego, CA, USA) and anti-T cadherin antibody (AnaSpec, Fremont, CA, USA). Immunoprecipitation was repeated on two separate occasions.

Furthermore, we extracted the plasma membrane of HRMEC, and performed the immunoprecipitation by use of that to confirm the interaction between T-cadherin and BiP in the cell membrane. The extraction of plasma membrane was performed by using Plasma Membrane Protein Extraction Kit (BioVision, Milpitas, CA) according to the manufacturer's protocol. After the operation, we performed by Western blotting using anti-VEGF receptor-2 antibody (Cell Signaling Technology, Beverly, MA) to determine the extraction of the plasma membrane of HRMEC. Next, immunocomplexes were made with the Pierce® Classic IP Kit with anti-T cadherin antibody (AnaSpec), immunoprecipitation products were analyzed by Western blotting using anti-BiP antibody (Abcam, Cambridge, UK), or by the Coomassie-staining (Wako).

### Immunofluorescence Staining of Non-permeabilized Cells

HRMEC were plated at the same density of proliferation onto 4-well collagen-coated LabTek-II chamber slides (Thermo Fisher Scientific), and incubated for 24 h at 37°C in 5% CO_2_. After preincubated in CSC medium containing 10% FBS without cell boost at 37°C for 24 h, HRMEC were supplemented with tunicamycin (10 ng/ml) for 2 h. Cells were washed twice with PBS and cell surface immunostaining was performed as previously described [43]. In brief, HRMEC were fixed with 4% PFA for 10 min, and then blocked with 10% horse serum in PBS for 30 min at room temperature. They were incubated in anti-T-cadherin antibody (1∶100) for 12 h at 4°C, rinsed with PBS, and further incubated in anti-BiP antibody (Santa Cruz Biotechnology, CA, USA) (1∶500) for 12 h at 4°C. After extensive washes, cells were stained with secondary antibodies diluted 1∶1000 in blocking solution and applied for 1 h at room temperature, all steps were without permeabilization. Confocal images of immunolabeled cells were pictured *via* confocal laser microscopy (FluoView FV10i; Olympus, Tokyo, Japan). All images had a thickness of 0.3 µm.

### Murine Oxygen-induced Retinopathy (OIR) Model

OIR model was produced as previously described by Smith et al [27]. At postnatal day 7 (P7), mice and their mothers were placed in a custom-built chamber and exposed to an atmosphere of almost 75% oxygen for 5 days. Oxygen was continuously monitored with an oxygen controller (PRO-OX 110; Reming Bioinstruments Co., Redfield, SD, USA). On P12, they were returned to room air until P17.

### Intravitreal Injection

Mice in OIR model or under normoxia were anesthetized with isoflurane and a 33-gauge needle was used to inject 1 µl of 3 µg/ml tunicamycin dissolved in 0.03% dimethyl sulfoxide (DMSO) in PBS or 0.03% DMSO in PBS (vehicle) into the vitreous of the eye under an operating microscope at P14. Thapsigargin at 1 µM dissolved in 0.01% DMSO in PBS or 0.01% DMSO in PBS was similarly injected into the vitreous of the eyes in mice.

To investigate the role of BiP for the retinal neovascularization, we examined with BiP inducer X (BiX) or BiP siRNA in the murine OIR model. BIX (10 µM, 1 µL) or vehicle (5% DMSO in PBS, 1 µL) were administered at P14 into the vitreous of the eyes in mice, as our previous report [26]. On the other hand, BiP siRNA (1 µg/mL, 1 µL) or vehicle (control siRNA, 1 µg/mL, 1 µL) were administered at P12. RNAi was achieved using a cocktail of two commercially available modified siRNAs, specific for BiP (Silencer siRNA #s67083; Invitrogen Inc., Carlsbad, CA, USA). A scrambled siRNA equivalent, not homologous to any known gene, served as a negative control, (#12935-300; Invitrogen Inc.). Before administration, siRNAs were encapsulated using a cationic liposome-based formulation (Invivofectamine; #1377–901; Invitrogen Inc.) in accordance with the manufacturer's instructions.

### Visualization of the Retinal Whole Mount by Angiography

Mice were deeply anesthetized intraperitoneally with sodium pentobarbital (Nembutal; Dainippon-Sumitomo Pharmaceutical Co. Ltd., Osaka, Japan) at 30 mg/kg. They were perfused through the left ventricle with fluorescein isothiocyanate (FITC)-conjugated dextran (Sigma Chemical, St. Louis, MO, USA) dissolved in PBS. Then, the eyes were enucleated and placed in 4% PFA. Under a microscope, the cornea and lens were removed from each eye, and the retinas were dissected, flat-mounted and covered with a coverslip after a few drops of Vectashield^®^ mounting median (Vector Laboratories, Burlingame, CA, USA) had been placed on the slide.

### Imaging and Quantification of Neovascularization

As described in our previous reports [44], [45], total images of flat-mounted retinas were produced *via* Metamorph (Universal Imaging Corp., Downingtown, PA, USA). To evaluate pathological neovascularization, we quantified the abnormal vasculature in the retina with the Angiogenesis Tube Formation module. In the quantification, the number of nodes and node areas were evaluated, which is parameters obtained from these analyzed images. The node is the region containing connected blobs with thickness exceeding a maximum width of the vessels and represents an area where there is pooling of FITC dextran. These regions are shown as green labels in analyzed images and corresponded well to the pathological neovascularization area (including tortuous and dilated blood vessels, and abnormal vascular structure). To evaluate neovasculature in the retina, the capillary-free area was also measured.

### The Assessment of the Localization of BiP in a Murine OIR Model retina

Eyes from OIR model mice and normal mice, perfused through the left ventricle with PBS or FITC-dextran, were fixed in 4% PFA at 4°C. For immunostaining of retinal flat-mounts, a small incision at the limbus of the enucleated eye was made to cut away the cornea and iris and then fine tip forceps were used to peel away the sclera, leaving the retina around the lens. Retinas were washed with TBS and treated with 0.01% trypsin for 15 min at room temperature. After blocked with 10% horse serum in PBS for 3 h, they were incubated in goat anti-BiP antibody (Santa Cruz Biotechnology) solution for 42 h at 4°C. Regarding retinas which perfused with PBS, they were further incubated with anti-CD31 antibody (AnaSpec) after extensive washing in TBS. Second antibodies were applied to retinas and they were flat-mounted under a coverslip. The sections and whole retinas were observed *via* FluoView FV10i (Olympus).

To detect the interaction between retinal neovascularization and BiP, we observed the retinal neovascularization and retinal BiP expression time-dependently by using the OIR model mice. After the OIR model mice at P12, P14, and P17 were perfused FITC-conjugated dextran, the left eyes were used as the analysis of retinal neovascularization as noted above, on the other hand, the right eyes were done as the sample for the detection of BiP expression. BiP expression was analyzed by Western blotting. Their eyeballs were quickly removed and the retinas were carefully separated from the eyeballs and frozen in dry ice. For protein extraction, the tissue was homogenized in cell-lysis buffer using a homogenizer (Physcotron, Microtec Co. Ltd., Chiba, Japan). The lysate was centrifuged at 12,000 g for 20 min, and the supernatant was used for this study. The protein concentrations were measured by comparison with a known concentration of bovine serum albumine using the BCA Protein Assay Kit (Thermo Fisher Scientific). A mixture of equal parts of an aliquot of protein and sample buffer with 10% 2-mercaptoethanol was subjected to 5%–20% sodium dodecyl sulfatepolyacrylamide gel electrophoresis. The separated protein was then transferred onto a polyvinylidene difluoride membrane (Immobilon-P; Millipore Billerica, MA, USA). For immunoblotting, the following primary antibodies were used BiP rabbit polyclonal antibody (Abcam) (1∶400) and β-actin mouse monoclonal antibody (Sigma-Aldrich) (1∶10000). Either goat anti-rabbit or anti-mouse horseradish peroxidase-conjugated (1∶2000) was used as a secondary antibody. The immunoreactive bands were visualized using ImmunoStar LD (Wako, Osaka, Japan), and measured using LAS-4000 (Fujifilm, Tokyo, Japan).

### Statistical Analysis

Data are presented as means ± S.E.M. Statistical comparisons were made using Student's *t*-test or Dunnett's multiple comparison test. *, p<0.05 and **, p<0.01 were considered statistically significant.
